# Supplementary material for: ‘Skeletal Age’ for mapping the impact of fracture on mortality
Source: eLife. 2023 May 16;12:e83888. doi: 10.7554/eLife.83888 (PMC10188111; doi:10.7554/eLife.83888)
Supplement: Supplementary file 1. [file elife-83888-supp1.docx]

**“Skeletal Age” for mapping the impact of fracture on mortality**

**Supplementary File 1. List of ICD-10 codes used to define specific fractures and comorbidities**

| **Conditions** | **ICD-10 codes** |
| --- | --- |
| **Fractures:** |  |
| *Proximal fractures:* |  |
| Hip | S72.0- S72.2 |
| Femur | S72.3-S72.9 |
| Pelvis | S32.3-S32.5 |
| Vertebrae | S22.0, S22.1, S32.0, S32.2, S32.7, S32.8, T08.x |
| Humerus | S42.x |
| Rib | S22.3-S22.4 |
| Clavicle | S42.0 |
| *Distal fractures:* |  |
| Forearm | S52.x |
| Lower leg | S82.2-S82.8 |
| Knee | S82.0 |
| Ankle | S82.5-S82.6 |
| Foot | S92.0-S92.3, S92.7, S92.9 |
| Hand | S62.0-S62.4, S62.8 |
| **Comorbidities:** |  |
| Myocardial infarct | I21.x, I22.x, I25.2 |
| Congestive heart failure | I09.9, I11.0, I13.0, I13.2, I25.5, I42.0, I42.5 - I42.9, I43.x, I50.x, P29.0 |
| Peripheral vascular disease | I70.x, I71.x, I73.1, I73.8, I73.9, I77.1, I79.0, I79.2, K55.1, K55.8, K55.9, Z95.8, Z95.9 |
| Cerebrovascular disease | G45.x, G46.x, H34.0, I60.x - I69.x |
| Cardiac valvular disease | A52.0, I05.x - I08.x, I09.1, I09.8, I34.x - I39.x, Q23.0 - Q23.3, Z95.2 - Z95.4 |
| Cardiac arrhythmias | I44.1 - I44.3, I45.6, I45.9, I47.x - I49.x, R00.0, R00.1, R00.8, T82.1, Z45.0, Z95.0 |
| Diabetes without chronic complication | E10.0, E10.1, E10.6, E10.8, E10.9, E11.0, E11.1, E11.6, E11.8, E11.9, E12.0, E12.1, E12.6, E12.8, E12.9, E13.0, E13.1, E13.6, E13.8, E13.9, E14.0, E14.1, E14.6, E14.8, E14.9 |
| Diabetes with chronic complication | E10.2 - E10.5, E10.7, E11.2 - E11.5, E11.7, E12.2 - E12.5, E12.7, E13.2 - E13.5, E13.7, E14.2 - E14.5, E14.7 |
| Any malignancy, including lymphoma and leukaemia, except malignant neoplasm of skin | C00.x - C26.x, C30.x - C34.x, C37.x - C41.x, C43.x, C45.x - C58.x, C60.x - C76.x, C81.x - C85.x, C88.x, C90.x - C97.x |
| Metastatic solid tumour | C77.x - C80.x |
| Rheumatic/Rheumatoid arthritis or collagen vascular disease | L94.0, L94.1, L94.3, M05.x, M06.x, M31.5, M32.x - M34.x, M08.x, M12.0, M12.3, M30.x, M31.0 - M31.3, M32.x - M35.x, M36.0, M45.x, M46.1, M46.8, M46.9 |
| Mild liver disease | B18.x, K70.0 - K70.3, K70.9, K71.3 - K71.5, K71.7, K73.x, K74.x, K76.0, K76.2 - K76.4, K76.8, K76.9, Z94.4 |
| Moderate or severe liver disease | I85.0, I85.9, I86.4, I98.2, K70.4, K71.1, K72.1, K72.9, K76.5, K76.6, K76.7 |
| Hypertension | I10.x, I11.x - I13.x, I15.x |
| Chronic pulmonary disease | I27.8, I27.9, J40.x - J47.x, J60.x - J67.x, J68.4, J70.1, J70.3 |
| Pulmonary circulation disorders | I26.x, I27.x, I28.0, I28.8, I28.9 |
| Dementia | F00.x - F03.x, F05.1, G30.x, G31.1 |
| Psychoses | F20.x, F22.x - F25.x, F28.x, F29.x, F30.2, F31.2, F31.5 |
| Depression | F20.4, F31.3 - F31.5, F32.x, F33.x, F34.1, F41.2, F43.2 |
| Neurological disorders | G10.x - G13.x, G20.x - G22.x, G25.4, G25.5, G31.2, G31.8, G31.9, G32.x, G35.x - G37.x, G40.x, G41.x, G93.1, G93.4, R47.0, R56.x |
| Hemiplegia or paraplegia | G04.1, G11.4, G80.1, G80.2, G81.x, G82.x, G83.0 - G83.4, G83.9 |
| Peptic ulcer disease | K25.x - K28.x |
| Chronic kidney disease | I12.0, I13.1, N03.2 - N03.7, N05.2 - N05.7, N18.x, N19.x, N25.0, Z49.0 - Z49.2, Z94.0, Z99.2 |
| Hypothyroidism | E00.x - E03.x, E89.0 |
| Coagulopathy | D65 - D68.x, D69.1, D69.3 - D69.6 |
| Obesity | E66.x |
| Weight loss | E40.x - E46.x, R63.4, R64 |
| Fluid and electrolyte disorders | E22.2, E86.x, E87.x |
| Anaemia | D50.0, D50.8, D50.9, D51.x - D53.x |
| Alcohol abuse | F10, E52, G62.1, I42.6, K29.2, K70.0, K70.3, K70.9, T51.x, Z50.2, Z71.4, Z72.1 |
| Drug abuse | F11.x - F16.x, F18.x, F19.x, Z71.5, Z72.2 |
| AIDS/HIV | B20.x - B22.x, B24.x |

**Table S2: Skeletal age by specific fracture site and chronological age at fracture**

| **Chronological age (years)** | **Any fracture** | **Hip fracture** | **Femur fracture** | **Pelvis fracture** | **Vertebral fracture** | **Humerus fracture** | **Rib fracture** | **Clavicle fracture** | **Lower leg fracture** |
| --- | --- | --- | --- | --- | --- | --- | --- | --- | --- |
| **Men** |  |  |  |  |  |  |  |  |  |
| 50 | 54.2 | 56.8 | 56.3 | 55.7 | 54.9 | 55.0 | 52.8 | 53.6 | 52.7 |
| 51 | 55.2 | 57.8 | 57.3 | 56.7 | 56.0 | 56.0 | 53.9 | 54.6 | 53.7 |
| 52 | 56.2 | 58.8 | 58.3 | 57.7 | 56.9 | 57.0 | 54.9 | 55.6 | 54.7 |
| 53 | 57.2 | 59.7 | 59.2 | 58.7 | 57.9 | 58.0 | 55.9 | 56.6 | 55.7 |
| 54 | 58.1 | 60.6 | 60.1 | 59.6 | 58.8 | 58.9 | 56.8 | 57.5 | 56.6 |
| 55 | 59.1 | 61.5 | 61.1 | 60.5 | 59.8 | 59.8 | 57.8 | 58.5 | 57.6 |
| 56 | 60.0 | 62.5 | 62 | 61.5 | 60.8 | 60.8 | 58.8 | 59.5 | 58.6 |
| 57 | 61.0 | 63.4 | 62.9 | 62.4 | 61.7 | 61.7 | 59.7 | 60.5 | 59.6 |
| 58 | 61.9 | 64.3 | 63.9 | 63.3 | 62.6 | 62.7 | 60.7 | 61.4 | 60.5 |
| 59 | 62.9 | 65.2 | 64.8 | 64.2 | 63.6 | 63.6 | 61.6 | 62.4 | 61.5 |
| 60 | 63.8 | 66.1 | 65.7 | 65.2 | 64.5 | 64.5 | 62.6 | 63.3 | 62.4 |
| 61 | 64.8 | 67.1 | 66.6 | 66.1 | 65.5 | 65.5 | 63.6 | 64.3 | 63.5 |
| 62 | 65.7 | 67.9 | 67.5 | 67.0 | 66.4 | 66.4 | 64.6 | 65.2 | 64.4 |
| 63 | 66.6 | 68.8 | 68.4 | 67.9 | 67.3 | 67.3 | 65.5 | 66.2 | 65.3 |
| 64 | 67.6 | 69.7 | 69.4 | 68.9 | 68.2 | 68.3 | 66.5 | 67.1 | 66.3 |
| 65 | 68.6 | 70.7 | 70.3 | 69.8 | 69.2 | 69.2 | 67.5 | 68.1 | 67.3 |
| 66 | 69.5 | 71.5 | 71.1 | 70.7 | 70.1 | 70.1 | 68.4 | 69.0 | 68.2 |
| 67 | 70.4 | 72.4 | 72.0 | 71.6 | 71.0 | 71.0 | 69.3 | 69.9 | 69.2 |
| 68 | 71.4 | 73.3 | 73.0 | 72.5 | 72.0 | 72.0 | 70.3 | 71.0 | 70.2 |
| 69 | 72.3 | 74.2 | 73.8 | 73.4 | 72.9 | 72.9 | 71.3 | 71.9 | 71.1 |
| 70 | 73.2 | 75.0 | 74.7 | 74.3 | 73.7 | 73.8 | 72.2 | 72.8 | 72.1 |
| 71 | 74.1 | 75.8 | 75.5 | 75.1 | 74.6 | 74.6 | 73.1 | 73.7 | 73.0 |
| 72 | 75.1 | 76.8 | 76.5 | 76.1 | 75.6 | 75.6 | 74.1 | 74.7 | 74.0 |
| 73 | 75.9 | 77.6 | 77.3 | 76.9 | 76.4 | 76.4 | 75.0 | 75.5 | 74.9 |
| 74 | 76.9 | 78.5 | 78.2 | 77.8 | 77.3 | 77.4 | 76.0 | 76.5 | 75.9 |
| 75 | 77.8 | 79.3 | 79.0 | 78.7 | 78.2 | 78.3 | 76.9 | 77.4 | 76.8 |
| 76 | 78.7 | 80.2 | 80.0 | 79.6 | 79.2 | 79.2 | 77.9 | 78.4 | 77.8 |
| 77 | 79.6 | 81.1 | 80.8 | 80.5 | 80.1 | 80.1 | 78.8 | 79.3 | 78.7 |
| 78 | 80.5 | 81.9 | 81.6 | 81.3 | 80.9 | 81.0 | 79.8 | 80.2 | 79.7 |
| 79 | 81.5 | 82.8 | 82.6 | 82.3 | 81.9 | 81.9 | 80.8 | 81.2 | 80.6 |
| 80 | 82.3 | 83.6 | 83.3 | 83.1 | 82.7 | 82.7 | 81.6 | 82.0 | 81.5 |
| 81 | 83.3 | 84.5 | 84.3 | 84 | 83.7 | 83.7 | 82.6 | 83.0 | 82.5 |
| 82 | 84.2 | 85.3 | 85.1 | 84.9 | 84.5 | 84.6 | 83.6 | 83.9 | 83.5 |
| 83 | 85.0 | 86.1 | 85.9 | 85.7 | 85.4 | 85.4 | 84.4 | 84.8 | 84.3 |
| 84 | 86.0 | 87.0 | 86.8 | 86.6 | 86.3 | 86.3 | 85.4 | 85.7 | 85.3 |
| 85 | 86.9 | 87.8 | 87.7 | 87.5 | 87.2 | 87.2 | 86.3 | 86.7 | 86.3 |
| 86 | 87.8 | 88.7 | 88.6 | 88.4 | 88.1 | 88.1 | 87.3 | 87.6 | 87.2 |
| 87 | 88.7 | 89.5 | 89.4 | 89.2 | 88.9 | 89.0 | 88.2 | 88.5 | 88.1 |
| 88 | 89.6 | 90.4 | 90.3 | 90.1 | 89.9 | 89.9 | 89.1 | 89.4 | 89.1 |
| 89 | 90.6 | 91.3 | 91.2 | 91.1 | 90.8 | 90.8 | 90.2 | 90.4 | 90.1 |
| 90 | 91.5 | 92.2 | 92.1 | 92.0 | 91.7 | 91.8 | 91.1 | 91.4 | 91.0 |
| 91 | 92.4 | 93.0 | 92.9 | 92.8 | 92.6 | 92.6 | 92.0 | 92.2 | 91.9 |
| 92 | 93.4 | 94.0 | 93.9 | 93.8 | 93.6 | 93.6 | 93.0 | 93.2 | 93.0 |
| 93 | 94.3 | 94.8 | 94.7 | 94.6 | 94.4 | 94.4 | 93.9 | 94.1 | 93.8 |
| 94 | 95.2 | 95.7 | 95.7 | 95.5 | 95.4 | 95.4 | 94.9 | 95.1 | 94.8 |
| 95 | 96.2 | 96.7 | 96.6 | 96.5 | 96.3 | 96.3 | 95.9 | 96.0 | 95.8 |
| 96 | 97.1 | 97.6 | 97.5 | 97.4 | 97.3 | 97.3 | 96.8 | 97.0 | 96.8 |
| 97 | 98.0 | 98.5 | 98.4 | 98.3 | 98.2 | 98.2 | 97.8 | 97.9 | 97.7 |
| 98 | 99.0 | 99.4 | 99.4 | 99.3 | 99.1 | 99.1 | 98.7 | 98.9 | 98.6 |
| 99 | 100.0 | 100.5 | 100.4 | 100.3 | 100.1 | 100.2 | 99.7 | 99.9 | 99.7 |
| 100 | 100.9 | 101.4 | 101.3 | 101.2 | 101.1 | 101.1 | 100.7 | 100.8 | 100.6 |
| **Women** |  |  |  |  |  |  |  |  |  |
| 50 | 53.3 | 55.3 | 55.3 | 54.4 | 54.4 | 52.6 | 52.7 | 53.2 | 52.0 |
| 51 | 54.3 | 56.3 | 56.4 | 55.4 | 55.4 | 53.6 | 53.8 | 54.3 | 53.1 |
| 52 | 55.3 | 57.2 | 57.3 | 56.3 | 56.3 | 54.5 | 54.7 | 55.2 | 54.0 |
| 53 | 56.3 | 58.3 | 58.3 | 57.4 | 57.4 | 55.6 | 55.7 | 56.2 | 55.0 |
| 54 | 57.3 | 59.2 | 59.3 | 58.3 | 58.3 | 56.6 | 56.8 | 57.2 | 56.1 |
| 55 | 58.2 | 60.1 | 60.2 | 59.2 | 59.2 | 57.5 | 57.7 | 58.1 | 57.0 |
| 56 | 59.2 | 61.1 | 61.1 | 60.2 | 60.2 | 58.5 | 58.7 | 59.1 | 58.0 |
| 57 | 60.2 | 62.1 | 62.1 | 61.2 | 61.2 | 59.5 | 59.7 | 60.1 | 59.0 |
| 58 | 61.2 | 63.1 | 63.1 | 62.2 | 62.2 | 60.5 | 60.7 | 61.1 | 60.0 |
| 59 | 62.1 | 63.9 | 64.0 | 63.1 | 63.1 | 61.4 | 61.6 | 62.0 | 60.9 |
| 60 | 63.1 | 64.9 | 64.9 | 64.1 | 64.1 | 62.4 | 62.6 | 63.0 | 61.9 |
| 61 | 64.1 | 65.9 | 65.9 | 65.0 | 65.0 | 63.4 | 63.6 | 64.0 | 62.9 |
| 62 | 65.0 | 66.8 | 66.8 | 66.0 | 66.0 | 64.4 | 64.5 | 65.0 | 63.9 |
| 63 | 66.0 | 67.7 | 67.8 | 66.9 | 66.9 | 65.3 | 65.5 | 65.9 | 64.8 |
| 64 | 66.9 | 68.6 | 68.7 | 67.8 | 67.8 | 66.3 | 66.4 | 66.9 | 65.8 |
| 65 | 67.9 | 69.6 | 69.7 | 68.9 | 68.9 | 67.3 | 67.5 | 67.9 | 66.9 |
| 66 | 68.9 | 70.5 | 70.6 | 69.8 | 69.8 | 68.2 | 68.4 | 68.8 | 67.8 |
| 67 | 69.8 | 71.4 | 71.5 | 70.7 | 70.7 | 69.2 | 69.3 | 69.7 | 68.7 |
| 68 | 70.8 | 72.4 | 72.4 | 71.7 | 71.7 | 70.2 | 70.3 | 70.7 | 69.8 |
| 69 | 71.7 | 73.3 | 73.3 | 72.6 | 72.6 | 71.1 | 71.3 | 71.7 | 70.7 |
| 70 | 72.7 | 74.2 | 74.2 | 73.5 | 73.5 | 72.1 | 72.2 | 72.6 | 71.7 |
| 71 | 73.7 | 75.2 | 75.2 | 74.5 | 74.5 | 73.1 | 73.2 | 73.6 | 72.7 |
| 72 | 74.6 | 76.1 | 76.1 | 75.4 | 75.4 | 74.0 | 74.2 | 74.5 | 73.6 |
| 73 | 75.5 | 76.9 | 77.0 | 76.3 | 76.3 | 75.0 | 75.1 | 75.5 | 74.6 |
| 74 | 76.4 | 77.8 | 77.8 | 77.2 | 77.2 | 75.9 | 76.0 | 76.4 | 75.5 |
| 75 | 77.4 | 78.7 | 78.8 | 78.1 | 78.1 | 76.9 | 77.0 | 77.4 | 76.5 |
| 76 | 78.3 | 79.6 | 79.6 | 79.0 | 79.0 | 77.8 | 77.9 | 78.3 | 77.5 |
| 77 | 79.3 | 80.6 | 80.6 | 80.0 | 80.0 | 78.8 | 79.0 | 79.3 | 78.5 |
| 78 | 80.3 | 81.5 | 81.5 | 80.9 | 80.9 | 79.8 | 79.9 | 80.2 | 79.4 |
| 79 | 81.2 | 82.4 | 82.4 | 81.8 | 81.8 | 80.7 | 80.8 | 81.2 | 80.4 |
| 80 | 82.0 | 83.1 | 83.2 | 82.6 | 82.6 | 81.6 | 81.7 | 82.0 | 81.3 |
| 81 | 83.0 | 84.1 | 84.1 | 83.6 | 83.6 | 82.6 | 82.7 | 83.0 | 82.3 |
| 82 | 83.9 | 84.9 | 85.0 | 84.5 | 84.5 | 83.5 | 83.6 | 83.9 | 83.2 |
| 83 | 84.8 | 85.8 | 85.8 | 85.4 | 85.4 | 84.5 | 84.5 | 84.8 | 84.2 |
| 84 | 85.7 | 86.7 | 86.7 | 86.3 | 86.3 | 85.4 | 85.5 | 85.7 | 85.1 |
| 85 | 86.7 | 87.6 | 87.6 | 87.2 | 87.2 | 86.4 | 86.4 | 86.7 | 86.1 |
| 86 | 87.7 | 88.5 | 88.5 | 88.1 | 88.1 | 87.3 | 87.4 | 87.6 | 87.1 |
| 87 | 88.6 | 89.4 | 89.4 | 89.0 | 89.0 | 88.2 | 88.3 | 88.5 | 88.0 |
| 88 | 89.5 | 90.2 | 90.3 | 89.9 | 89.9 | 89.2 | 89.3 | 89.5 | 89.0 |
| 89 | 90.4 | 91.1 | 91.1 | 90.8 | 90.8 | 90.1 | 90.2 | 90.4 | 89.9 |
| 90 | 91.3 | 92.0 | 92.0 | 91.7 | 91.7 | 91.0 | 91.1 | 91.3 | 90.8 |
| 91 | 92.2 | 92.8 | 92.8 | 92.6 | 92.6 | 92.0 | 92.0 | 92.2 | 91.8 |
| 92 | 93.2 | 93.8 | 93.8 | 93.6 | 93.6 | 93.0 | 93.1 | 93.2 | 92.8 |
| 93 | 94.1 | 94.7 | 94.7 | 94.4 | 94.4 | 93.9 | 93.9 | 94.1 | 93.7 |
| 94 | 95.1 | 95.6 | 95.6 | 95.3 | 95.3 | 94.8 | 94.9 | 95.0 | 94.7 |
| 95 | 96.0 | 96.5 | 96.5 | 96.3 | 96.3 | 95.8 | 95.9 | 96.0 | 95.7 |
| 96 | 96.9 | 97.4 | 97.4 | 97.2 | 97.2 | 96.7 | 96.8 | 96.9 | 96.6 |
| 97 | 97.9 | 98.4 | 98.4 | 98.2 | 98.2 | 97.7 | 97.8 | 97.9 | 97.6 |
| 98 | 98.8 | 99.3 | 99.3 | 99.1 | 99.1 | 98.7 | 98.7 | 98.8 | 98.5 |
| 99 | 99.8 | 100.2 | 100.2 | 100.0 | 100.0 | 99.6 | 99.7 | 99.8 | 99.5 |
| 100 | 100.7 | 101.1 | 101.1 | 100.9 | 100.9 | 100.6 | 100.6 | 100.7 | 100.4 |

**Table S3: Skeletal age for a 60-year-old individual who sustained a fracture at a specific bone**

| **Fracture** | **Skeletal age in years (95% CI)** | |
| --- | --- | --- |
|  | **Men** | **Women** |
| Any fragility fracture | 63.8 (63.7, 63.9) | 63.1 (63.0, 63.2) |
| Specific fracture site |  |  |
| Hip | 66.1 (65.9, 66.2) | 64.9 (64.8, 65.0) |
| Femur | 65.7 (65.2, 66.1) | 64.9 (64.6, 65.2) |
| Pelvis | 65.2 (64.6, 65.8) | 64.1 (63.8, 64.4) |
| Vertebrae | 64.5 (64.2, 64.7) | 64.1 (63.9, 64.3) |
| Humerus | 64.5 (64.2, 64.8) | 62.4 (62.3, 62.6) |
| Rib | 62.6 (62.2, 63.1) | 62.6 (62.2, 63.0) |
| Clavicle | 63.3 (62.9, 63.7) | 63.0 (62.6, 63.4) |
| Lower leg | 62.4 (61.8, 62.8) | 61.9 (61.6, 62.2) |

**Table S4. R codes used to construct skeletal age for individual fracture sites associated with increased mortality risk**

**# (1) Skeletal age for the high-risk fracture sites in men**

***## Analysis macro***

func_Skel_Age <- function(sk.age, gender, fracture, thres_age){

qx = c( 2.059, 0.175, 0.204, 0.033, 0.064, 0.032, 0.096, 0.094, 0.000, 0.000, 0.000, 0.000, 0.060, 0.151, 0.153, 0.185, 0.369, 0.437, 0.589, 0.445, 0.577, 0.579, 0.317, 0.476, 0.441, 0.713, 0.895, 0.477, 0.543, 0.611, 0.507, 0.638, 0.572, 0.740, 0.849, 0.620, 0.840, 0.729, 0.960, 0.771, 0.906, 1.306, 1.020, 1.419, 1.055, 1.160, 1.448, 1.782, 1.712, 2.112, 1.873, 2.174, 2.949, 2.198, 2.617, 3.138, 4.276, 4.323, 4.333, 5.031, 5.880, 5.611, 6.368, 7.896, 7.888, 8.288, 11.187, 12.640, 12.769, 13.729, 15.648, 16.632, 18.592, 20.907, 24.536, 27.730, 28.554, 30.961, 36.073, 41.158, 47.602, 51.960, 61.761, 66.589, 78.630, 92.359, 100.496, 112.117, 128.829, 151.725, 162.987, 179.113, 204.320, 222.943, 235.269, 245.015, 294.884, 340.168, 345.695, 350.626, 364.807, 350.383, 338.862, 283.469, 550.671, 435.282, 1000.000)

age = 0:106

fx_hr = sk.age$est[sk.age$sex == gender & sk.age$fx == fracture]

Lx1 = qx

lx1 = qx

Tx1 = qx

Ex1 = qx

Lx2 = qx

lx2 = qx

Tx2 = qx

Ex2 = qx

length = length(qx)-1

Lx1[1] = 100000

Lx1[length+1] = NA

Tx1[length+1] = NA

Ex1[length+1] = NA

Lx2[1] = 100000

Lx2[length+1] = NA

Tx2[length+1] = NA

Ex2[length+1] = NA

for (x in 2:length) {

Lx1[x] = Lx1[x-1]*(1-(qx[x-1]*1.00)/1000)

Lx2[x] = Lx2[x-1]*(1-(qx[x-1]*fx_hr)/1000)

}

lx1 = (Lx1 + lead(Lx1))/2

lx1[length] = 0

lx1[length+1] = NA

lx2 = (Lx2 + lead(Lx2))/2

lx2[length] = 0

lx2[length+1] = NA

for (x in 1:length) {

Tx1[x] = sum(lx1[x:length])

Tx2[x] = sum(lx2[x:length])

}

Ex1 = Tx1 / Lx1

Ex2 = Tx2 / Lx2

Lx1 = round(Lx1)

lx1 = round(lx1)

Tx1 = round(Tx1)

Ex1 = round(Ex1,1)

Lx2 = round(Lx2)

lx2 = round(lx2)

Tx2 = round(Tx2)

Ex2 = round(Ex2,1)

Diff = Ex1- Ex2

Skel.Age = age+Diff

print(Skel.Age)

df = data.frame(age, qx, Ex1, Ex2, Diff, Skel.Age)

df$fx = fracture

skage = subset(df, age>thres_age, select = c(age, fx, Skel.Age))

skage

}

***## Dataset***

sk.age = read.csv("C:\\Garvan\\Skeletal age\\Analysis\\Skeletal_age.csv")

***## Analysis***

*### (1.1) Any fracture*

any_fx_men = func_Skel_Age(sk.age, "Men", "Any fracture",49)

any_fx_men

*### (1.2) Hip fracture*

hip_fx_men = func_Skel_Age(sk.age, "Men", "Hip", 49)

hip_fx_men

*### (1.3) Femur fracture*

femur_fx_men = func_Skel_Age(sk.age, "Men", "Femur", 49)

femur_fx_men

*### (1.4) Pelvis fracture*

pelvis_fx_men = func_Skel_Age(sk.age, "Men", "Pelvis", 49)

pelvis_fx_men

*### (1.5) Vertebral fracture*

vert_fx_men = func_Skel_Age(sk.age, "Men", "Vertebrae", 49)

vert_fx_men

*### (1.6) Humerus fracture*

hum_fx_men = func_Skel_Age(sk.age, "Men", "Humerus", 49)

hum_fx_men

*### (1.7) Rib fracture*

rib_fx_men = func_Skel_Age(sk.age, "Men", "Rib", 49)

rib_fx_men

*### (1.8) Clavicle fracture*

clav_fx_men = func_Skel_Age(sk.age, "Men", "Clavicle", 49)

clav_fx_men

*### (1.9) Lower leg fracture*

leg_fx_men = func_Skel_Age(sk.age, "Men", "Lower leg", 49)

leg_fx_men

*### Dataset - Skeletal age for high-risk fracture sites in men*

library(dplyr)

library(tidyverse)

df_list = list(any_fx_men, hip_fx_men, femur_fx_men, pelvis_fx_men, vert_fx_men, hum_fx_men, rib_fx_men, clav_fx_men, leg_fx_men)

sa.men = df_list %>% reduce(full_join, by = "age")

head(sa.men)

**# (2) Skeletal age for the high-risk fracture sites in women**

***## Analysis macro***

func_Skel_Age <- function(sk.age, gender, fracture, thres_age){

qx = c(1.331, 0.148, 0.072, 0.035, 0.034, 0.034, 0.067, 0.131, 0.032, 0.063, 0.124, 0.124, 0.095, 0.191, 0.096, 0.162, 0.130, 0.294, 0.163, 0.063, 0.187, 0.218, 0.185, 0.210, 0.147, 0.320, 0.200, 0.251, 0.217, 0.370, 0.210, 0.317, 0.377, 0.304, 0.225, 0.398, 0.317, 0.433, 0.665, 0.523, 0.526, 0.709, 0.900, 0.630, 0.498, 0.705, 1.026, 1.067, 1.556, 1.456, 1.452, 1.313, 1.448, 2.205, 2.413, 2.365, 2.487, 2.633, 2.636, 2.993, 3.567, 3.901, 5.035, 5.070, 5.352, 6.482, 6.636, 7.310, 7.955, 9.231, 9.933, 12.446, 14.182, 15.618, 16.567, 16.675, 19.111, 23.939, 26.242, 27.700, 35.226, 34.403, 44.070, 50.708, 58.747, 66.721, 64.897, 81.356, 96.449, 114.857, 126.731, 137.871, 161.417, 180.891, 209.809, 229.592, 241.967, 282.213, 299.071, 322.506, 378.927, 330.566, 366.203, 400.171, 327.020, 329.680, 1000.000)

age = 0:106

fx_hr = sk.age$est[sk.age$sex == gender & sk.age$fx == fracture]

Lx1 = qx

lx1 = qx

Tx1 = qx

Ex1 = qx

Lx2 = qx

lx2 = qx

Tx2 = qx

Ex2 = qx

length = length(qx)-1

Lx1[1] = 100000

Lx1[length+1] = NA

Tx1[length+1] = NA

Ex1[length+1] = NA

Lx2[1] = 100000

Lx2[length+1] = NA

Tx2[length+1] = NA

Ex2[length+1] = NA

for (x in 2:length) {

Lx1[x] = Lx1[x-1]*(1-(qx[x-1]*1.00)/1000)

Lx2[x] = Lx2[x-1]*(1-(qx[x-1]*fx_hr)/1000)

}

lx1 = (Lx1 + lead(Lx1))/2

lx1[length] = 0

lx1[length+1] = NA

lx2 = (Lx2 + lead(Lx2))/2

lx2[length] = 0

lx2[length+1] = NA

for (x in 1:length) {

Tx1[x] = sum(lx1[x:length])

Tx2[x] = sum(lx2[x:length])

}

Ex1 = Tx1 / Lx1

Ex2 = Tx2 / Lx2

Lx1 = round(Lx1)

lx1 = round(lx1)

Tx1 = round(Tx1)

Ex1 = round(Ex1,1)

Lx2 = round(Lx2)

lx2 = round(lx2)

Tx2 = round(Tx2)

Ex2 = round(Ex2,1)

Diff = Ex1- Ex2

Skel.Age = age+Diff

print(Skel.Age)

df = data.frame(age, qx, Ex1, Ex2, Diff, Skel.Age)

df$fx = fracture

skage = subset(df, age>thres_age, select = c(age, fx, Skel.Age))

skage

}

***## Analysis***

*### (2.1) Any fracture*

any_fx_women = func_Skel_Age(sk.age, "Women", "Any fracture",49)

any_fx_women

*### (2.2) Hip fracture*

hip_fx_women = func_Skel_Age(sk.age, "Women", "Hip", 49)

hip_fx_women

*### (2.3) Femur fracture*

femur_fx_women = func_Skel_Age(sk.age, "Women", "Femur", 49)

femur_fx_women

*### (2.4) Pelvis fracture*

pelvis_fx_women = func_Skel_Age(sk.age, "Women", "Pelvis", 49)

pelvis_fx_women

*### (2.5) Vertebral fracture*

vert_fx_women = func_Skel_Age(sk.age, "Women", "Vertebrae", 49)

vert_fx_women

*### (2.6) Humerus fracture*

hum_fx_women = func_Skel_Age(sk.age, "Women", "Humerus", 49)

hum_fx_women

*### (2.7) Rib fracture*

rib_fx_women = func_Skel_Age(sk.age, "Women", "Rib", 49)

rib_fx_women

*### (2.8) Clavicle fracture*

clav_fx_women = func_Skel_Age(sk.age, "Women", "Clavicle", 49)

clav_fx_women

*### (2.9) Lower leg fracture*

leg_fx_women = func_Skel_Age(sk.age, "Women", "Lower leg", 49)

leg_fx_women

*### Dataset - Skeletal age for high-risk fracture sites in women*

df_list = list(any_fx_women, hip_fx_women, femur_fx_women, pelvis_fx_women, vert_fx_women, hum_fx_women, rib_fx_women, clav_fx_women, leg_fx_women)

sa.women = df_list %>% reduce(full_join, by = "age")

head(sa.women)

***## Dataset for both men and women***

sa.both = rbind(sa.men, sa.women)

sa.both

write.csv(sa.both, "C:\\Garvan\\Skeletal age\\Analysis\\Skeletal_age_both.csv", row.names = FALSE)
