# Supplementary material for: ‘Skeletal Age’ for mapping the impact of fracture on mortality
Source: eLife. 2023 May 16;12:e83888. doi: 10.7554/eLife.83888 (PMC10188111; doi:10.7554/eLife.83888)
Supplement: Supplementary file 2. [file elife-83888-supp2.docx]

**“Skeletal Age” for mapping the impact of fracture on mortality**

**Supplementary File 2. Skeletal age by specific fracture site and chronological age at fracture**

| **Chronological age (years)** | **Any fracture** | **Hip fracture** | **Femur fracture** | **Pelvis fracture** | **Vertebral fracture** | **Humerus fracture** | **Rib fracture** | **Clavicle fracture** | **Lower leg fracture** |
| --- | --- | --- | --- | --- | --- | --- | --- | --- | --- |
| **Men** |  |  |  |  |  |  |  |  |  |
| 50 | 54.2 | 56.8 | 56.3 | 55.7 | 54.9 | 55.0 | 52.8 | 53.6 | 52.7 |
| 51 | 55.2 | 57.8 | 57.3 | 56.7 | 56.0 | 56.0 | 53.9 | 54.6 | 53.7 |
| 52 | 56.2 | 58.8 | 58.3 | 57.7 | 56.9 | 57.0 | 54.9 | 55.6 | 54.7 |
| 53 | 57.2 | 59.7 | 59.2 | 58.7 | 57.9 | 58.0 | 55.9 | 56.6 | 55.7 |
| 54 | 58.1 | 60.6 | 60.1 | 59.6 | 58.8 | 58.9 | 56.8 | 57.5 | 56.6 |
| 55 | 59.1 | 61.5 | 61.1 | 60.5 | 59.8 | 59.8 | 57.8 | 58.5 | 57.6 |
| 56 | 60.0 | 62.5 | 62 | 61.5 | 60.8 | 60.8 | 58.8 | 59.5 | 58.6 |
| 57 | 61.0 | 63.4 | 62.9 | 62.4 | 61.7 | 61.7 | 59.7 | 60.5 | 59.6 |
| 58 | 61.9 | 64.3 | 63.9 | 63.3 | 62.6 | 62.7 | 60.7 | 61.4 | 60.5 |
| 59 | 62.9 | 65.2 | 64.8 | 64.2 | 63.6 | 63.6 | 61.6 | 62.4 | 61.5 |
| 60 | 63.8 | 66.1 | 65.7 | 65.2 | 64.5 | 64.5 | 62.6 | 63.3 | 62.4 |
| 61 | 64.8 | 67.1 | 66.6 | 66.1 | 65.5 | 65.5 | 63.6 | 64.3 | 63.5 |
| 62 | 65.7 | 67.9 | 67.5 | 67.0 | 66.4 | 66.4 | 64.6 | 65.2 | 64.4 |
| 63 | 66.6 | 68.8 | 68.4 | 67.9 | 67.3 | 67.3 | 65.5 | 66.2 | 65.3 |
| 64 | 67.6 | 69.7 | 69.4 | 68.9 | 68.2 | 68.3 | 66.5 | 67.1 | 66.3 |
| 65 | 68.6 | 70.7 | 70.3 | 69.8 | 69.2 | 69.2 | 67.5 | 68.1 | 67.3 |
| 66 | 69.5 | 71.5 | 71.1 | 70.7 | 70.1 | 70.1 | 68.4 | 69.0 | 68.2 |
| 67 | 70.4 | 72.4 | 72.0 | 71.6 | 71.0 | 71.0 | 69.3 | 69.9 | 69.2 |
| 68 | 71.4 | 73.3 | 73.0 | 72.5 | 72.0 | 72.0 | 70.3 | 71.0 | 70.2 |
| 69 | 72.3 | 74.2 | 73.8 | 73.4 | 72.9 | 72.9 | 71.3 | 71.9 | 71.1 |
| 70 | 73.2 | 75.0 | 74.7 | 74.3 | 73.7 | 73.8 | 72.2 | 72.8 | 72.1 |
| 71 | 74.1 | 75.8 | 75.5 | 75.1 | 74.6 | 74.6 | 73.1 | 73.7 | 73.0 |
| 72 | 75.1 | 76.8 | 76.5 | 76.1 | 75.6 | 75.6 | 74.1 | 74.7 | 74.0 |
| 73 | 75.9 | 77.6 | 77.3 | 76.9 | 76.4 | 76.4 | 75.0 | 75.5 | 74.9 |
| 74 | 76.9 | 78.5 | 78.2 | 77.8 | 77.3 | 77.4 | 76.0 | 76.5 | 75.9 |
| 75 | 77.8 | 79.3 | 79.0 | 78.7 | 78.2 | 78.3 | 76.9 | 77.4 | 76.8 |
| 76 | 78.7 | 80.2 | 80.0 | 79.6 | 79.2 | 79.2 | 77.9 | 78.4 | 77.8 |
| 77 | 79.6 | 81.1 | 80.8 | 80.5 | 80.1 | 80.1 | 78.8 | 79.3 | 78.7 |
| 78 | 80.5 | 81.9 | 81.6 | 81.3 | 80.9 | 81.0 | 79.8 | 80.2 | 79.7 |
| 79 | 81.5 | 82.8 | 82.6 | 82.3 | 81.9 | 81.9 | 80.8 | 81.2 | 80.6 |
| 80 | 82.3 | 83.6 | 83.3 | 83.1 | 82.7 | 82.7 | 81.6 | 82.0 | 81.5 |
| 81 | 83.3 | 84.5 | 84.3 | 84 | 83.7 | 83.7 | 82.6 | 83.0 | 82.5 |
| 82 | 84.2 | 85.3 | 85.1 | 84.9 | 84.5 | 84.6 | 83.6 | 83.9 | 83.5 |
| 83 | 85.0 | 86.1 | 85.9 | 85.7 | 85.4 | 85.4 | 84.4 | 84.8 | 84.3 |
| 84 | 86.0 | 87.0 | 86.8 | 86.6 | 86.3 | 86.3 | 85.4 | 85.7 | 85.3 |
| 85 | 86.9 | 87.8 | 87.7 | 87.5 | 87.2 | 87.2 | 86.3 | 86.7 | 86.3 |
| 86 | 87.8 | 88.7 | 88.6 | 88.4 | 88.1 | 88.1 | 87.3 | 87.6 | 87.2 |
| 87 | 88.7 | 89.5 | 89.4 | 89.2 | 88.9 | 89.0 | 88.2 | 88.5 | 88.1 |
| 88 | 89.6 | 90.4 | 90.3 | 90.1 | 89.9 | 89.9 | 89.1 | 89.4 | 89.1 |
| 89 | 90.6 | 91.3 | 91.2 | 91.1 | 90.8 | 90.8 | 90.2 | 90.4 | 90.1 |
| 90 | 91.5 | 92.2 | 92.1 | 92.0 | 91.7 | 91.8 | 91.1 | 91.4 | 91.0 |
| 91 | 92.4 | 93.0 | 92.9 | 92.8 | 92.6 | 92.6 | 92.0 | 92.2 | 91.9 |
| 92 | 93.4 | 94.0 | 93.9 | 93.8 | 93.6 | 93.6 | 93.0 | 93.2 | 93.0 |
| 93 | 94.3 | 94.8 | 94.7 | 94.6 | 94.4 | 94.4 | 93.9 | 94.1 | 93.8 |
| 94 | 95.2 | 95.7 | 95.7 | 95.5 | 95.4 | 95.4 | 94.9 | 95.1 | 94.8 |
| 95 | 96.2 | 96.7 | 96.6 | 96.5 | 96.3 | 96.3 | 95.9 | 96.0 | 95.8 |
| 96 | 97.1 | 97.6 | 97.5 | 97.4 | 97.3 | 97.3 | 96.8 | 97.0 | 96.8 |
| 97 | 98.0 | 98.5 | 98.4 | 98.3 | 98.2 | 98.2 | 97.8 | 97.9 | 97.7 |
| 98 | 99.0 | 99.4 | 99.4 | 99.3 | 99.1 | 99.1 | 98.7 | 98.9 | 98.6 |
| 99 | 100.0 | 100.5 | 100.4 | 100.3 | 100.1 | 100.2 | 99.7 | 99.9 | 99.7 |
| 100 | 100.9 | 101.4 | 101.3 | 101.2 | 101.1 | 101.1 | 100.7 | 100.8 | 100.6 |
| **Women** |  |  |  |  |  |  |  |  |  |
| 50 | 53.3 | 55.3 | 55.3 | 54.4 | 54.4 | 52.6 | 52.7 | 53.2 | 52.0 |
| 51 | 54.3 | 56.3 | 56.4 | 55.4 | 55.4 | 53.6 | 53.8 | 54.3 | 53.1 |
| 52 | 55.3 | 57.2 | 57.3 | 56.3 | 56.3 | 54.5 | 54.7 | 55.2 | 54.0 |
| 53 | 56.3 | 58.3 | 58.3 | 57.4 | 57.4 | 55.6 | 55.7 | 56.2 | 55.0 |
| 54 | 57.3 | 59.2 | 59.3 | 58.3 | 58.3 | 56.6 | 56.8 | 57.2 | 56.1 |
| 55 | 58.2 | 60.1 | 60.2 | 59.2 | 59.2 | 57.5 | 57.7 | 58.1 | 57.0 |
| 56 | 59.2 | 61.1 | 61.1 | 60.2 | 60.2 | 58.5 | 58.7 | 59.1 | 58.0 |
| 57 | 60.2 | 62.1 | 62.1 | 61.2 | 61.2 | 59.5 | 59.7 | 60.1 | 59.0 |
| 58 | 61.2 | 63.1 | 63.1 | 62.2 | 62.2 | 60.5 | 60.7 | 61.1 | 60.0 |
| 59 | 62.1 | 63.9 | 64.0 | 63.1 | 63.1 | 61.4 | 61.6 | 62.0 | 60.9 |
| 60 | 63.1 | 64.9 | 64.9 | 64.1 | 64.1 | 62.4 | 62.6 | 63.0 | 61.9 |
| 61 | 64.1 | 65.9 | 65.9 | 65.0 | 65.0 | 63.4 | 63.6 | 64.0 | 62.9 |
| 62 | 65.0 | 66.8 | 66.8 | 66.0 | 66.0 | 64.4 | 64.5 | 65.0 | 63.9 |
| 63 | 66.0 | 67.7 | 67.8 | 66.9 | 66.9 | 65.3 | 65.5 | 65.9 | 64.8 |
| 64 | 66.9 | 68.6 | 68.7 | 67.8 | 67.8 | 66.3 | 66.4 | 66.9 | 65.8 |
| 65 | 67.9 | 69.6 | 69.7 | 68.9 | 68.9 | 67.3 | 67.5 | 67.9 | 66.9 |
| 66 | 68.9 | 70.5 | 70.6 | 69.8 | 69.8 | 68.2 | 68.4 | 68.8 | 67.8 |
| 67 | 69.8 | 71.4 | 71.5 | 70.7 | 70.7 | 69.2 | 69.3 | 69.7 | 68.7 |
| 68 | 70.8 | 72.4 | 72.4 | 71.7 | 71.7 | 70.2 | 70.3 | 70.7 | 69.8 |
| 69 | 71.7 | 73.3 | 73.3 | 72.6 | 72.6 | 71.1 | 71.3 | 71.7 | 70.7 |
| 70 | 72.7 | 74.2 | 74.2 | 73.5 | 73.5 | 72.1 | 72.2 | 72.6 | 71.7 |
| 71 | 73.7 | 75.2 | 75.2 | 74.5 | 74.5 | 73.1 | 73.2 | 73.6 | 72.7 |
| 72 | 74.6 | 76.1 | 76.1 | 75.4 | 75.4 | 74.0 | 74.2 | 74.5 | 73.6 |
| 73 | 75.5 | 76.9 | 77.0 | 76.3 | 76.3 | 75.0 | 75.1 | 75.5 | 74.6 |
| 74 | 76.4 | 77.8 | 77.8 | 77.2 | 77.2 | 75.9 | 76.0 | 76.4 | 75.5 |
| 75 | 77.4 | 78.7 | 78.8 | 78.1 | 78.1 | 76.9 | 77.0 | 77.4 | 76.5 |
| 76 | 78.3 | 79.6 | 79.6 | 79.0 | 79.0 | 77.8 | 77.9 | 78.3 | 77.5 |
| 77 | 79.3 | 80.6 | 80.6 | 80.0 | 80.0 | 78.8 | 79.0 | 79.3 | 78.5 |
| 78 | 80.3 | 81.5 | 81.5 | 80.9 | 80.9 | 79.8 | 79.9 | 80.2 | 79.4 |
| 79 | 81.2 | 82.4 | 82.4 | 81.8 | 81.8 | 80.7 | 80.8 | 81.2 | 80.4 |
| 80 | 82.0 | 83.1 | 83.2 | 82.6 | 82.6 | 81.6 | 81.7 | 82.0 | 81.3 |
| 81 | 83.0 | 84.1 | 84.1 | 83.6 | 83.6 | 82.6 | 82.7 | 83.0 | 82.3 |
| 82 | 83.9 | 84.9 | 85.0 | 84.5 | 84.5 | 83.5 | 83.6 | 83.9 | 83.2 |
| 83 | 84.8 | 85.8 | 85.8 | 85.4 | 85.4 | 84.5 | 84.5 | 84.8 | 84.2 |
| 84 | 85.7 | 86.7 | 86.7 | 86.3 | 86.3 | 85.4 | 85.5 | 85.7 | 85.1 |
| 85 | 86.7 | 87.6 | 87.6 | 87.2 | 87.2 | 86.4 | 86.4 | 86.7 | 86.1 |
| 86 | 87.7 | 88.5 | 88.5 | 88.1 | 88.1 | 87.3 | 87.4 | 87.6 | 87.1 |
| 87 | 88.6 | 89.4 | 89.4 | 89.0 | 89.0 | 88.2 | 88.3 | 88.5 | 88.0 |
| 88 | 89.5 | 90.2 | 90.3 | 89.9 | 89.9 | 89.2 | 89.3 | 89.5 | 89.0 |
| 89 | 90.4 | 91.1 | 91.1 | 90.8 | 90.8 | 90.1 | 90.2 | 90.4 | 89.9 |
| 90 | 91.3 | 92.0 | 92.0 | 91.7 | 91.7 | 91.0 | 91.1 | 91.3 | 90.8 |
| 91 | 92.2 | 92.8 | 92.8 | 92.6 | 92.6 | 92.0 | 92.0 | 92.2 | 91.8 |
| 92 | 93.2 | 93.8 | 93.8 | 93.6 | 93.6 | 93.0 | 93.1 | 93.2 | 92.8 |
| 93 | 94.1 | 94.7 | 94.7 | 94.4 | 94.4 | 93.9 | 93.9 | 94.1 | 93.7 |
| 94 | 95.1 | 95.6 | 95.6 | 95.3 | 95.3 | 94.8 | 94.9 | 95.0 | 94.7 |
| 95 | 96.0 | 96.5 | 96.5 | 96.3 | 96.3 | 95.8 | 95.9 | 96.0 | 95.7 |
| 96 | 96.9 | 97.4 | 97.4 | 97.2 | 97.2 | 96.7 | 96.8 | 96.9 | 96.6 |
| 97 | 97.9 | 98.4 | 98.4 | 98.2 | 98.2 | 97.7 | 97.8 | 97.9 | 97.6 |
| 98 | 98.8 | 99.3 | 99.3 | 99.1 | 99.1 | 98.7 | 98.7 | 98.8 | 98.5 |
| 99 | 99.8 | 100.2 | 100.2 | 100.0 | 100.0 | 99.6 | 99.7 | 99.8 | 99.5 |
| 100 | 100.7 | 101.1 | 101.1 | 100.9 | 100.9 | 100.6 | 100.6 | 100.7 | 100.4 |
