# Supplementary material for: ‘Skeletal Age’ for mapping the impact of fracture on mortality
Source: eLife. 2023 May 16;12:e83888. doi: 10.7554/eLife.83888 (PMC10188111; doi:10.7554/eLife.83888)
Supplement: Supplementary file 3. [file elife-83888-supp3.docx]

**“Skeletal Age” for mapping the impact of fracture on mortality**

**Supplementary File 3. Skeletal age for a 60-year-old individual who sustained a fracture at a specific bone**

| **Fracture** | **Skeletal age in years (95% CI)** | |
| --- | --- | --- |
|  | **Men** | **Women** |
| Any fragility fracture | 63.8 (63.7, 63.9) | 63.1 (63.0, 63.2) |
| Specific fracture site |  |  |
| Hip | 66.1 (65.9, 66.2) | 64.9 (64.8, 65.0) |
| Femur | 65.7 (65.2, 66.1) | 64.9 (64.6, 65.2) |
| Pelvis | 65.2 (64.6, 65.8) | 64.1 (63.8, 64.4) |
| Vertebrae | 64.5 (64.2, 64.7) | 64.1 (63.9, 64.3) |
| Humerus | 64.5 (64.2, 64.8) | 62.4 (62.3, 62.6) |
| Rib | 62.6 (62.2, 63.1) | 62.6 (62.2, 63.0) |
| Clavicle | 63.3 (62.9, 63.7) | 63.0 (62.6, 63.4) |
| Lower leg | 62.4 (61.8, 62.8) | 61.9 (61.6, 62.2) |
