# Supplementary material for: ‘Skeletal Age’ for mapping the impact of fracture on mortality
Source: eLife. 2023 May 16;12:e83888. doi: 10.7554/eLife.83888 (PMC10188111; doi:10.7554/eLife.83888)
Supplement: Supplementary file 4. [file elife-83888-supp4.docx]

**“Skeletal Age” for mapping the impact of fracture on mortality**

**Supplementary File 4. R codes used to construct skeletal age for individual fracture sites associated with increased mortality risk**

**# (1) Skeletal age for the high-risk fracture sites in men**

***## Analysis macro***

func_Skel_Age <- function(sk.age, gender, fracture, thres_age){

qx = c( 2.059, 0.175, 0.204, 0.033, 0.064, 0.032, 0.096, 0.094, 0.000, 0.000, 0.000, 0.000, 0.060, 0.151, 0.153, 0.185, 0.369, 0.437, 0.589, 0.445, 0.577, 0.579, 0.317, 0.476, 0.441, 0.713, 0.895, 0.477, 0.543, 0.611, 0.507, 0.638, 0.572, 0.740, 0.849, 0.620, 0.840, 0.729, 0.960, 0.771, 0.906, 1.306, 1.020, 1.419, 1.055, 1.160, 1.448, 1.782, 1.712, 2.112, 1.873, 2.174, 2.949, 2.198, 2.617, 3.138, 4.276, 4.323, 4.333, 5.031, 5.880, 5.611, 6.368, 7.896, 7.888, 8.288, 11.187, 12.640, 12.769, 13.729, 15.648, 16.632, 18.592, 20.907, 24.536, 27.730, 28.554, 30.961, 36.073, 41.158, 47.602, 51.960, 61.761, 66.589, 78.630, 92.359, 100.496, 112.117, 128.829, 151.725, 162.987, 179.113, 204.320, 222.943, 235.269, 245.015, 294.884, 340.168, 345.695, 350.626, 364.807, 350.383, 338.862, 283.469, 550.671, 435.282, 1000.000)

age = 0:106

fx_hr = sk.age$est[sk.age$sex == gender & sk.age$fx == fracture]

Lx1 = qx

lx1 = qx

Tx1 = qx

Ex1 = qx

Lx2 = qx

lx2 = qx

Tx2 = qx

Ex2 = qx

length = length(qx)-1

Lx1[1] = 100000

Lx1[length+1] = NA

Tx1[length+1] = NA

Ex1[length+1] = NA

Lx2[1] = 100000

Lx2[length+1] = NA

Tx2[length+1] = NA

Ex2[length+1] = NA

for (x in 2:length) {

Lx1[x] = Lx1[x-1]*(1-(qx[x-1]*1.00)/1000)

Lx2[x] = Lx2[x-1]*(1-(qx[x-1]*fx_hr)/1000)

}

lx1 = (Lx1 + lead(Lx1))/2

lx1[length] = 0

lx1[length+1] = NA

lx2 = (Lx2 + lead(Lx2))/2

lx2[length] = 0

lx2[length+1] = NA

for (x in 1:length) {

Tx1[x] = sum(lx1[x:length])

Tx2[x] = sum(lx2[x:length])

}

Ex1 = Tx1 / Lx1

Ex2 = Tx2 / Lx2

Lx1 = round(Lx1)

lx1 = round(lx1)

Tx1 = round(Tx1)

Ex1 = round(Ex1,1)

Lx2 = round(Lx2)

lx2 = round(lx2)

Tx2 = round(Tx2)

Ex2 = round(Ex2,1)

Diff = Ex1- Ex2

Skel.Age = age+Diff

print(Skel.Age)

df = data.frame(age, qx, Ex1, Ex2, Diff, Skel.Age)

df$fx = fracture

skage = subset(df, age>thres_age, select = c(age, fx, Skel.Age))

skage

}

***## Dataset***

sk.age = read.csv("C:\\Garvan\\Skeletal age\\Analysis\\Skeletal_age.csv")

***## Analysis***

*### (1.1) Any fracture*

any_fx_men = func_Skel_Age(sk.age, "Men", "Any fracture",49)

any_fx_men

*### (1.2) Hip fracture*

hip_fx_men = func_Skel_Age(sk.age, "Men", "Hip", 49)

hip_fx_men

*### (1.3) Femur fracture*

femur_fx_men = func_Skel_Age(sk.age, "Men", "Femur", 49)

femur_fx_men

*### (1.4) Pelvis fracture*

pelvis_fx_men = func_Skel_Age(sk.age, "Men", "Pelvis", 49)

pelvis_fx_men

*### (1.5) Vertebral fracture*

vert_fx_men = func_Skel_Age(sk.age, "Men", "Vertebrae", 49)

vert_fx_men

*### (1.6) Humerus fracture*

hum_fx_men = func_Skel_Age(sk.age, "Men", "Humerus", 49)

hum_fx_men

*### (1.7) Rib fracture*

rib_fx_men = func_Skel_Age(sk.age, "Men", "Rib", 49)

rib_fx_men

*### (1.8) Clavicle fracture*

clav_fx_men = func_Skel_Age(sk.age, "Men", "Clavicle", 49)

clav_fx_men

*### (1.9) Lower leg fracture*

leg_fx_men = func_Skel_Age(sk.age, "Men", "Lower leg", 49)

leg_fx_men

*### Dataset - Skeletal age for high-risk fracture sites in men*

library(dplyr)

library(tidyverse)

df_list = list(any_fx_men, hip_fx_men, femur_fx_men, pelvis_fx_men, vert_fx_men, hum_fx_men, rib_fx_men, clav_fx_men, leg_fx_men)

sa.men = df_list %>% reduce(full_join, by = "age")

head(sa.men)

**# (2) Skeletal age for the high-risk fracture sites in women**

***## Analysis macro***

func_Skel_Age <- function(sk.age, gender, fracture, thres_age){

qx = c(1.331, 0.148, 0.072, 0.035, 0.034, 0.034, 0.067, 0.131, 0.032, 0.063, 0.124, 0.124, 0.095, 0.191, 0.096, 0.162, 0.130, 0.294, 0.163, 0.063, 0.187, 0.218, 0.185, 0.210, 0.147, 0.320, 0.200, 0.251, 0.217, 0.370, 0.210, 0.317, 0.377, 0.304, 0.225, 0.398, 0.317, 0.433, 0.665, 0.523, 0.526, 0.709, 0.900, 0.630, 0.498, 0.705, 1.026, 1.067, 1.556, 1.456, 1.452, 1.313, 1.448, 2.205, 2.413, 2.365, 2.487, 2.633, 2.636, 2.993, 3.567, 3.901, 5.035, 5.070, 5.352, 6.482, 6.636, 7.310, 7.955, 9.231, 9.933, 12.446, 14.182, 15.618, 16.567, 16.675, 19.111, 23.939, 26.242, 27.700, 35.226, 34.403, 44.070, 50.708, 58.747, 66.721, 64.897, 81.356, 96.449, 114.857, 126.731, 137.871, 161.417, 180.891, 209.809, 229.592, 241.967, 282.213, 299.071, 322.506, 378.927, 330.566, 366.203, 400.171, 327.020, 329.680, 1000.000)

age = 0:106

fx_hr = sk.age$est[sk.age$sex == gender & sk.age$fx == fracture]

Lx1 = qx

lx1 = qx

Tx1 = qx

Ex1 = qx

Lx2 = qx

lx2 = qx

Tx2 = qx

Ex2 = qx

length = length(qx)-1

Lx1[1] = 100000

Lx1[length+1] = NA

Tx1[length+1] = NA

Ex1[length+1] = NA

Lx2[1] = 100000

Lx2[length+1] = NA

Tx2[length+1] = NA

Ex2[length+1] = NA

for (x in 2:length) {

Lx1[x] = Lx1[x-1]*(1-(qx[x-1]*1.00)/1000)

Lx2[x] = Lx2[x-1]*(1-(qx[x-1]*fx_hr)/1000)

}

lx1 = (Lx1 + lead(Lx1))/2

lx1[length] = 0

lx1[length+1] = NA

lx2 = (Lx2 + lead(Lx2))/2

lx2[length] = 0

lx2[length+1] = NA

for (x in 1:length) {

Tx1[x] = sum(lx1[x:length])

Tx2[x] = sum(lx2[x:length])

}

Ex1 = Tx1 / Lx1

Ex2 = Tx2 / Lx2

Lx1 = round(Lx1)

lx1 = round(lx1)

Tx1 = round(Tx1)

Ex1 = round(Ex1,1)

Lx2 = round(Lx2)

lx2 = round(lx2)

Tx2 = round(Tx2)

Ex2 = round(Ex2,1)

Diff = Ex1- Ex2

Skel.Age = age+Diff

print(Skel.Age)

df = data.frame(age, qx, Ex1, Ex2, Diff, Skel.Age)

df$fx = fracture

skage = subset(df, age>thres_age, select = c(age, fx, Skel.Age))

skage

}

***## Analysis***

*### (2.1) Any fracture*

any_fx_women = func_Skel_Age(sk.age, "Women", "Any fracture",49)

any_fx_women

*### (2.2) Hip fracture*

hip_fx_women = func_Skel_Age(sk.age, "Women", "Hip", 49)

hip_fx_women

*### (2.3) Femur fracture*

femur_fx_women = func_Skel_Age(sk.age, "Women", "Femur", 49)

femur_fx_women

*### (2.4) Pelvis fracture*

pelvis_fx_women = func_Skel_Age(sk.age, "Women", "Pelvis", 49)

pelvis_fx_women

*### (2.5) Vertebral fracture*

vert_fx_women = func_Skel_Age(sk.age, "Women", "Vertebrae", 49)

vert_fx_women

*### (2.6) Humerus fracture*

hum_fx_women = func_Skel_Age(sk.age, "Women", "Humerus", 49)

hum_fx_women

*### (2.7) Rib fracture*

rib_fx_women = func_Skel_Age(sk.age, "Women", "Rib", 49)

rib_fx_women

*### (2.8) Clavicle fracture*

clav_fx_women = func_Skel_Age(sk.age, "Women", "Clavicle", 49)

clav_fx_women

*### (2.9) Lower leg fracture*

leg_fx_women = func_Skel_Age(sk.age, "Women", "Lower leg", 49)

leg_fx_women

*### Dataset - Skeletal age for high-risk fracture sites in women*

df_list = list(any_fx_women, hip_fx_women, femur_fx_women, pelvis_fx_women, vert_fx_women, hum_fx_women, rib_fx_women, clav_fx_women, leg_fx_women)

sa.women = df_list %>% reduce(full_join, by = "age")

head(sa.women)

***## Dataset for both men and women***

sa.both = rbind(sa.men, sa.women)

sa.both

write.csv(sa.both, "C:\\Garvan\\Skeletal age\\Analysis\\Skeletal_age_both.csv", row.names = FALSE)
